# Enterprise financialization and technological innovation: Mechanism and heterogeneity

**DOI:** 10.1371/journal.pone.0275461

**Published:** 2022-12-12

**Authors:** Yue Liu, Pierre Failler, Yan Ding

**Affiliations:** 1 Business School, Hunan Institute of Technology, Hengyang, China; 2 Economics and Finance Group, Portsmouth Business School, University of Portsmouth, Portsmouth, United Kingdom; Bucharest University of Economic Studies: Academia de Studii Economice din Bucuresti, ROMANIA

## Abstract

After the 2008 financial crisis, under the double effects of enterprise value maximization and the decline of real economy marginal profit, the relationship between enterprise financialization and technological innovation is worth exploring in depth. On the basis of testing the impact of non-financial enterprise financialization on technological innovation, this paper explores the impact mechanism as well as the heterogeneity among different types of enterprises. This paper selects non-financial listed enterprises in China from 2007 to 2017 as samples to study the influence of enterprise financialization on technological innovation and its mechanism through panel regression and mediating effect models. Moreover, the heterogeneity among different types of enterprises is further studied. The main conclusions are as follows. First, the financialization of enterprises has a significant "crowding out" effect on technological innovation. Second, the “crowding out” effect of enterprise financialization on technological innovation is formed through capital structure rather than performance. Third, enterprises are faced with different attributes and external environment, thus the influence of financialization on technological innovation is heterogeneous. Fourth, there are significant differences in the impact of financialization on technological innovation between enterprises’ attributes and the external environment they face, and the deviation degree caused by attributes is much greater than that caused by the external environment.

## Introduction

Controversial conclusions are drawn about the relationship between financialization and technological innovation by analyzing the financialization behavior of non-financial enterprises from both macro and micro perspectives. From the perspective of macro-financial development, it is believed that financialization plays a positive role in enterprise R & D innovation. Because capital investment is an endogenous variable of enterprise technological innovation. Enterprise financialization can broaden financing channels, improve financing efficiency, ease financing constraints, and increase the capital supply of technological innovation [1–4]. But excessive financialization will make the industrial focus shift from the real economy sector to the virtual economy sector, forming the industry hollowing-out, which weakens the foundation of technological innovation [5]. Literature analyzed from the micro level of enterprises believes that the financialization behavior of enterprises has negative effects, because in the process of a continuous flow of industrial capital to the financial sector, the increase of financial asset allocation and the improvement of short-term financial investment returns further strengthen the inductive effect of financial investment of enterprises [6]. Ohangazi (2008) [7] found that non-financial enterprises gain more benefits from investment in financial assets and financial institutions, leading to a crowding-out effect on investment in corporate entities. Xie J. Z. et al. (2014) [8] believed that excessive financialization of the manufacturing industry will accelerate the "de-industrialization", weaken the development foundation of the manufacturing industry, and thus inhibit the innovation ability of enterprises. Chong L. et al. (2019) [9] found that with the deepening of financialization in the field of non-financial enterprises, even if enterprises have the ability to take risks, they have no willingness to do that, and they demonstrated theoretically the micro inducement for the lack of development motivation of enterprises under the "siphon effect" of financialization. Besides, business creators’ potential decision to launch social business ventures is positively influenced by technological propensity but also constrained mainly by lack of investment capital and scarce access to finance [10].

There are many typical examples of the contradiction between enterprise financialization and technological innovation in reality, making the negative relationship between the two reach a consensus to a certain extent. This contradiction is mainly due to the pursuit of short-term shareholder value maximization by non-financial enterprises, as well as the huge capital demand and uncertainty of technological innovation.

On the one hand, financialization reflects the pursuit of shareholder value maximization of non-financial enterprises. Due to the pursuit of short-term shareholder value maximization by enterprises, financial investment activities of non-financial enterprises are gradually active, and the proportion of financial investment and profits from financial channels are constantly increasing. The profit accumulation of non-financial enterprises increasingly relies on financial channels rather than traditional trade and commodity production [11]. Non-financial enterprises are involved in the financial market [12]. The core of various economic behaviors has changed from the production sector and some extended service sector to the financial sector. This series of enterprise financialization behaviors originate from the influence of financial practice and financial system development on corporate governance and shareholder value [13–16]. When non-financial enterprises allocate more assets to the financial sector, it will cause the macro-economy to "turn from real to virtual" [17].

On the other hand, the technological innovation of enterprises is often accompanied by huge capital demand and great uncertainty. Enterprise technological innovation is often accompanied by a huge demand for capital [18, 19]. When non-financial enterprises allocate more assets to the financial field, and when enterprise technological innovation lacks physical assets, it is generally difficult for them to obtain financial support, resulting in the lack of sources of technological innovation. At the same time, the uncertainty of technological innovation is very strong, which makes the risk of investment greater. According to Kor and Mahoney (2005) [20], the resource allocation of enterprises will affect their technological level. An enterprise with large R & D investment can provide a good development environment for technological innovation and obtain a sustainable competitive position. Yang (2019) [21] found that the financialization of real enterprises was negatively correlated with R&D investment in the current period and significantly positively correlated with R&D investment in the lag period, showing a cumulative effect. Guo L. (2017) [22] believes that manufacturing financialization has a crowding-out effect on enterprise innovation investment; however, with the continuous improvement of enterprise operating performance and the continuous easing of enterprise financing constraints, financialization has gradually presented a reservoir effect on innovation investment. Brown et al. (2012) [23] believe that enterprises can maintain the smooth development of R&D and innovation activities through cash reserves to avoid fluctuations in R&D and innovation due to cash flow. Tian and Wang (2014) [24] found that innovation requires more time and resources and has a lower chance of success, so the uncertainty brought by innovation is very large. Yong et al. (2020) [25] hold that with the deepening of financialization and the worsening of management myopia, economic entities will direct technological innovation strategies towards incremental innovation at the expense of fundamental innovation involving high risk and long-term investment.

The research on the relationship between enterprise financialization and technological innovation is far more than the discussion on the results of a positive or negative relationship. The key to solving the contradiction is to explore how enterprise financialization affects technological innovation and whether there is heterogeneity in different samples. Therefore, on the basis of verifying the significant impact of enterprise financialization on technological innovation, this paper systematically analyzes the mediating variables in the process of the impact and then studies the impact mechanism of financialization on technological innovation; in addition, based on the sub-sample test, this paper studies the heterogeneity of the impact of different enterprises’ financialization on technological innovation. The main work is as follows: First, this paper analyzes whether the financialization behavior of non-financial enterprises has a significant impact on technological innovation through econometric models. Second, the impact mechanism is systematically analyzed and the mediating variables in the process of action are dug out through experiments. Third, based on the sub-sample test, we study the heterogeneity of the financialization behavior of different types of enterprises on technological innovation.

Through the research, the main conclusions are drawn as follows. First, enterprise financialization has an inhibitory effect on technological innovation. Although there is still divergence on the relationship between financialization and technological innovation, this paper proves that the improvement of the level of enterprise financialization will inhibit technological innovation; at the same time, through the analysis of the impact mechanism of enterprise financialization on technological innovation, it is found that enterprise financialization mainly suppresses technological innovation through capital structure; the enterprise performance index, i.e. the net profit margin, has no mediating effect. Second, the impact of financialization behaviour on technological innovation is heterogeneous among enterprises with different internal attributes. By extracting the attribute characteristics of enterprises, it is found that the industry and ownership attributes of enterprises have significant differences in the effect on the enterprise financialization behavior. From the perspective of industry attributes, the financialization of enterprises in non-heavily polluting industries has a significant inhibitory effect on technological innovation; from the perspective of ownership attributes, except for state-owned enterprises and private enterprises, the financialization of other enterprises has a significant inhibitory effect on technological innovation. Third, the impact of financialization behaviour on technological innovation is heterogeneous among enterprises facing different external environments. Through the analysis of the external environment, it is found that the region where the enterprise is located and the financing constraints also have different impacts on the enterprise financialization behavior. From the perspective of the region where the enterprises are located, the financialization of the enterprises in the western region has a stronger inhibitory effect on technological innovation; from the perspective of financing constraints, the financialization of enterprises with low financing constraints has a stronger inhibitory effect on technological innovation. The selected two types of attribute characteristics and two kinds of external environment have a significant impact on enterprise technological innovation. The relationship between enterprise financialization and technological innovation is more sensitive to the difference in industry attributes.

The structure of the following parts of this paper is as follows. Firstly, the research scheme is proposed, and the overall impact of financialization behavior on technological innovation is tested through the econometric model. Secondly, we analyze which variables have the mediating effect in the influence of enterprise financialization on technological innovation, and the impact mechanism of enterprise financialization on technological innovation is further analyzed. Thirdly, the heterogeneity of the impact of financialization behavior on technological innovation is studied from the perspectives of the enterprise attribute characteristics and the external environment they face. The last part draws the main conclusion and propose some political implications.

## Research scheme

### Research hypothesis

Since the 2008 international financial crisis, stylized fact of investment substitution of non-financial enterprises has led to crowding-out effects of financialization on scientific and technological innovation. In developed countries, the profit margin of traditional productive industries has been declining for a long time, and emerging countries have seen insufficient demand and prominent structural problems, resulting in a sustained downturn in the real economy. At the same time, the rate of return on financial investment is rising. When the real economy is depressed, the government frequently launches loose monetary policies, and the excess liquidity flows into the capital market and the real estate market, resulting in asset price bubbles and real estate price bubbles. The alternately prosperous stock market and real estate market have led to the continuous rise of financial investment yield. The fact that the return rate of financial investment is higher than that of real economy investment strengthens the financialization tendency of non-financial enterprises when they pursue the goal of profit maximization, reducing their investment in technological innovation. In addition, the measurement of managers’ value and contribution to enterprises is usually accomplished through short-term performance indicators, such as loss reduction and profit decline [26]. Therefore, managers tend to choose projects with high returns and short periodicity to maximize shareholder value [27, 28]. At the same time, since innovation activities usually have the characteristics of long periodicity, unpredictable results, and high failure risks [29], enterprises usually start from the value of shareholders to reduce private costs rather than invest funds in technological innovation, and they tend to allocate financial assets in order to obtain short-term benefits, thus crowding out technological innovation. Based on this, this paper puts forward the following hypothesis.

**Hypothesis 1:** on the basis of the stylized fact, non-financial enterprises will reduce their investment in technological innovation, and their financialization behavior will produce a crowding-out effect on technological innovation.

The relationship between enterprise financialization and technological innovation may also be affected by other factors. From the perspective of the financial capital operation mechanism, when the goal of enterprise financialization is to maximize shareholder value, it tends to use the financial leverage effect to maximize the profit-seeking function of capital. The leverage ratio of enterprises means that the economic entities control large scales of assets with small capital by means of debt financing. The intervention of financial institutions can effectively alleviate adverse selection and moral hazards under information asymmetry, reduce the external financing cost of enterprise technological innovation, and promote technological innovation [30]. In addition, the earnings from the financialization behavior of enterprises can be used for the development of the main business to improve the net profit of enterprises, thus providing funds for technological innovation activities and promoting their realization. Based on this, this paper considers the enterprise capital structure and operating net profit rate as the mediating variables of financialization affecting technological innovation and puts forward the following hypothesis:

**Hypothesis 2:** capital structure and operating net profit rate play mediating roles in the impact mechanism of enterprise financialization on technological innovation.

When enterprises have different attributes or are in different external environments, the impact of financialization on technological innovation will be different. The characteristics of industry and ownership belong to the internal characteristics of enterprises, which are difficult to change in the short term. The enterprises’ attribute characteristics are relatively stable and objective, and they need to pay more cost when changing. The region where an enterprise is located and the financing constraints are external environmental factors, which are easier to change than the characteristics of enterprise attributes. Therefore, considering the internal characteristics and external environment of different enterprises, this paper further analyzes the heterogeneity of the impact of enterprise financialization on technological innovation and proposes the following hypothesis:

**Hypothesis 3:** from the perspective of enterprise attributes and the external environment, the impact of financialization on technological innovation is heterogeneous.

### Research methodology

The impact of enterprise financialization on technological innovation will show significant differences in different industries and different development stages. Enterprises belong to different industries, and their competitors and financial levels are different, leading to significant differences in the effect of financialization on technological innovation. For example, enterprises in technology-intensive competition industries have stronger requirements for advanced technology, stronger technical objectives, and more tendentious investment in technological innovation. In the time dimension, the difference in the enterprise development stage restricts the technological innovation investment in the process of asset allocation. When an enterprise is in the initial stage of its life cycle, it has a greater demand for liquid funds, a weaker ability to bear uncertain risks, and a stronger willingness to allocate liquid assets. When an enterprise is in the mature stage of its life cycle, it has a strong ability to bear uncertain risks, and it may be more inclined to invest in technological innovation for strategic consideration. Panel data models can investigate the impact of enterprise financialization on technological innovation from the dimensions of time and space, so this paper adopts the panel data model to conduct econometric tests. The basic form of the model set is as follows:

$$\text{LnApply}\_{\text{s}}_{\text{it}}=\beta_{0}+\beta_{1}{\text{Financialzation}}_{\text{it}}+\sum \alpha_{\text{i}}{\text{X}}_{\text{it}}+\varepsilon_{\text{it}},$$ (1)

where subscripts **i** and **t** denote enterprise and year, respectively. **LnApply_s** is the explained variable, which indicates the technological innovation level of enterprises. This paper uses the number of patent applications of listed companies and takes its natural logarithm as a measure of technological innovation activities. **Financialization** is an explanatory variable, representing the financialization behavior of enterprises. Referring to Demir (2009) [31], this paper uses the ratio of financial assets to total assets at the end of the period to measure the financialization behavior of enterprises. Financial assets include transaction financial assets, investment real estate, long-term financial equity investment and entrusted financial management, and trust products. After summing up, the total assets are used for standardization. In addition, in order to test the robustness of the model results, this paper selects an index (**Financialization 2**) to measure the level of enterprise financialization from the perspective of income to replace the proportion of financial assets in total assets for model tests. It is measured by the ratio of financial asset income to enterprise operating profit.

This paper introduces the relevant control variable **X** to control the impact of other characteristics of enterprises on the level of technological innovation. Because many factors affect the level of technological innovation, according to the relevant theory and existing empirical research [32, 33], this paper considers adding other related variables that affect the level of technological innovation in the process of modeling. In the study of the impact of enterprise financialization behavior on the level of technological innovation, it is necessary to assume that other factors remain unchanged, that is, other main factors need to be controlled and set as control variables in the econometric test. Combined with the characteristics of listed companies in China, this paper introduces a total of 7 control variables from the micro and macro levels, among which there are 4 control variables at the micro level, including operating net cash flow (CFO), enterprise size (Lnsize), enterprise capital intensive (Fixed), and enterprise age (Lnage); at the macro level, there are three control variables, including loan-to-deposit ratio (Loandep), industrial structure (Industr), and economic development level (LnperGDP) of the region where the enterprise is located.

At the same time, considering that the financialization behavior of enterprises may have an impact on the level of technological innovation of enterprises through their financial situation, this paper introduces the capital structure of enterprises and the net profit margin of enterprises as mediating variables. The names and measurement methods of the above variables are shown in Table 1.

**Table 1:** Mediating variables and control variables.

Variable type	Variable name	Measure method
**mediating variable**	the capital structure of enterprises (Lev)	ratio of total liabilities to total assets at the end of the period
	the net profit margin of enterprises (Roa)	ratio of net profit to total assets at the end of the period
**control variable**	operating net cash flow (CFO)	ratio of net cash flow from operating activities to total assets at the end of the period
	enterprise size (Lnsize)	natural logarithm of total assets at the end of the period
	enterprise capital intensive (Fixed)	ratio of fixed assets to total assets at the end of the period
	enterprise age (Lnage)	the current year minus the enterprise registration year plus 1 and take the natural logarithm
	loan-to-deposit ratio (Loandep)	ratio of domestic and foreign currency deposits and loans balance of financial institutions at the end of the year
	industrial structure (Industr)	ratio of added value of secondary industry to regional GDP
	economic development level (LnperGDP)	natural logarithm of regional per capita GDP

### Data source and description

From the perspective of sample scope, this paper selects non-financial enterprises of China’s A-share listed companies as the research object. The main reason is that, for financial enterprises, their business is to allocate and use financial assets, which is significantly different from financial asset allocation of non-financial assets. At the same time, enterprises with financial channel earnings or negative operating profits are excluded, because when the financial channel earnings index is used for the robustness test, the results of enterprise financialization are the same, but the contribution degree of enterprise financialization is significantly different. From the perspective of the sample time dimension, considering that the economic structure of both developed and developing countries has changed significantly after the international financial crisis in 2008, the typical characteristics of non-financial enterprises are more prominent, and the international financial crisis in 2008 began with the US subprime mortgage crisis in 2007, so the sample time dimension of this paper is set from 2007; according to the availability of data, the number of patent applications of Chinese listed companies is temporarily updated to 2017. Based on this, the sample time range of this paper is set from 2007 to 2017. Due to the lack of some indicators in individual years, the data in this paper belong to unbalanced panel data.

As for data sources, the data on technological innovation come from the State Intellectual Property Office of the People’s Republic of China; the data on financialization level and micro level control variables come from the Guotai’an CSMAR database, and the data of macro level control variables come from China’s regional financial operation report issued by the People’s Bank of China, EPS data platform, and the official website of the National Bureau of Statistics over the years. The year and region are matched to each object. In order to eliminate the possible influence of outliers on the robustness of regression results, we perform Winsorize processing of variables Financialization, Lev, CFO, Lnsize, and Fixed on the 1% and 99% percentiles. The statistics of all variables are shown in Table 2.

**Table 2:** Descriptive statistics of variables.

Variable	Obs	Mean	Std.Dev.	Min	Max
**lnApply_s**	18,036	2.1401	1.7253	0.0000	6.6241
**Financialization**	17,909	0.0746	0.1016	0.0000	0.5799
**Financialization 2**	18,036	0.1293	0.2093	0.0000	0.9995
**CFO**	17,664	0.0482	0.0687	-0.1835	0.2517
**Lnsize**	17,716	21.8879	1.1878	19.5363	25.8612
**Lnage**	18,033	2.7507	0.3516	0.0000	4.2195
**Fixed**	17,687	0.2214	0.1604	0.0023	0.7251
**loandep**	18,036	0.7067	0.1169	0.2328	1.1013
**industr**	18,036	0.4470	0.0904	0.1901	0.5905
**lnperGDP**	18,036	10.8703	0.5193	8.9718	11.7675
**Lev**	17,707	0.4144	0.2023	0.0474	0.8931
**Roa**	18,036	0.0495	0.0634	-2.7463	0.5900

**Fig 1:**
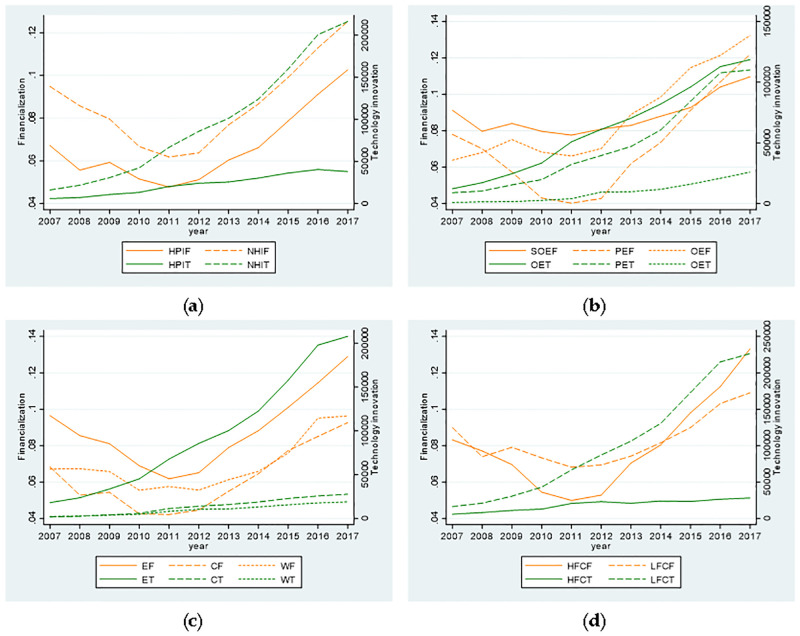
Line chart of the annual means of financialization degree, annual sums of technological innovation quantity and the years. **Notes: (a)** The line chart of industries with different pollution degrees. HPIF and NHIF represent the annual mean of the financialization degree of heavily polluting industries and non-heavily polluting industries, respectively. HPIT and NHIT represent the annual sum of the technological innovation quantity of heavily polluting industries and non-heavily polluting industries respectively. **(b)** The line chart of enterprises with different ownership. SOEF, PEF and OEF respectively represent the annual mean of financialization degree of state-owned enterprises, private enterprises and other enterprises; SOET, PET and OET respectively represent the annual sum of technological innovation quantity of state-owned enterprises, private enterprises and other enterprises. **(c)** The line chart of enterprises in different regions. EF, CF and WF represent the annual mean of financialization degree of enterprises in eastern, central and western regions, respectively. ET, CT and WT represent the annual sum of technological innovation quantity of enterprises in eastern, central and western regions, respectively. **(d)** The line chart of enterprises with different financing constraints. HFCF and LFCF represent the annual mean of financialization degree of enterprises with high and low financing constraints respectively, and HFCT and LFCT represent the annual sum of the number of technological innovation of enterprises with high and low financing constraints respectively.

Table 2 reports descriptive statistics for all variables. On the whole, the minimum value of enterprise technological innovation is 0.0000, the maximum value is 6.6241, and the average value is 2.1401, indicating that the overall level of enterprise technological innovation is not high. In terms of the financialization behavior of enterprises, the minimum value of financial assets is 0.0000, the maximum value is 0.5799, and the average value is 0.0663, indicating that different listed non-financial enterprises have different degrees of financialization. In addition, this paper also conducts descriptive statistics from four attributes of ownership, industry, location and financing constraints of enterprises, as shown in Fig 1.

It can be seen from Fig 1(a) that the financialization levels of enterprises in both heavily polluting industry and non-heavily polluting industry, on the whole, show a downward trend and then an upward trend, and the change range is basically the same, reaching the minimum around 2011. The technological innovation abilities of enterprises in both heavily polluting industry and non-heavily polluting industry are increasing with time. The technological innovation ability of enterprises in the non-heavily polluting industry is improving very fast, while that of heavily polluting industry is improving slowly.

In Fig 1(b), the financialization level of state-owned enterprises and other enterprises is on the rise as a whole; the financialization level of private enterprises first decreases and then increases with time, with a large range of changes, and it reaches the minimum value in 2011. Before 2013, the financialization level of state-owned enterprises is higher than that of other enterprises, but after 2013, the financialization level of other enterprises is higher than that of state-owned enterprises. For technological innovation ability, state-owned enterprises are the highest, followed by private enterprises, and other enterprises are the lowest. However, the technological innovation ability of these three types of enterprises has been improving over time. Among them, the improvement speed of state-owned enterprises and private enterprises is faster than that of other enterprises.

As can be seen from Fig 1(c), the financialization levels of enterprises in the eastern, central and western regions all show a trend of first declining and then rising. The financial level of enterprises in the east is the highest, followed by those in the west and those in the central part of China. For the technological innovation ability, the eastern enterprises are the strongest, the central enterprises are the second, and the western enterprises are the weakest. The technological innovation capability of enterprises in eastern China increases rapidly with time, while that of enterprises in central and western China increases slowly with time.

According to Fig 1(d), the financialization level of enterprises with both high and low financing constraints, on the whole, shows a trend of first decline and then rise. From 2007 to 2014, the financialization level of enterprises with low financing constraint is higher than that of enterprises with high financing constraints. After 2014, the financialization level of enterprises with high financing constraints exceeds that of enterprises with low financing constraints, and the financialization level of the two types of enterprises reaches the minimum value around 2011. In terms of the technological innovation ability, enterprises with low financing constraints significantly improve over time and are much stronger than those with high financing constraints.

## The impact mechanism of enterprise financialization on technological innovation

### Econometric test of the impact of enterprise financialization on technological innovation

Before analyzing the impact mechanism of enterprise financialization on technological innovation, Hypothesis 1 mentioned in the second part (research scheme part) needs to be tested. Firstly, the stability test of the relevant variables is carried out and relevant data are preprocessed. Then, the parameters of the benchmark model are estimated with the data. At the same time, the robustness test of parameter estimation results is carried out from the perspectives of variable substitution and estimation methods. From the perspective of variable substitution, the financialization level is constructed by using relevant accounting indicators obtained from income channels, and the benchmark model is re-used for estimation. From the perspective of estimation methods, based on the robustness test of the estimation method, the first-order lag term of capital intensity and the first-order lag term of the net profit margin of enterprises are used as instrumental variables to control the endogeneity of the model, and the GMM method is used for parameter estimation. The estimation results of model parameters are shown in Table 3.

**Table 3:** The estimation results of model parameters.

Variable	LnApply_s	LnApply_s	LnApply_s
	(1)	(2)	(3)
**Financialization**	-0.3694***		-22.8189***
	(0.0905)		(0.8611)
**Financialization2**		-0.0326**	
		(0.0141)	
**CFO**	0.2451*	0.1435	2.3500***
	(0.1396)	(0.1570)	(0.3147)
**Lnsize**	0.5021***	0.5046***	0.3468***
	(0.0091)	(0.0093)	(0.0173)
**Lnage**	-0.1373***	-0.0968***	0.8779***
	(0.0328)	(0.0341)	(0.0741)
**Fixed**	-1.5159***	-1.4489***	-3.7271***
	(0.0701)	(0.0756)	(0.1547)
**Loandep**	-0.3786***	-0.3560***	0.7533***
	(0.0961)	(0.1071)	(0.1953)
**Industr**	1.5551***	1.5277***	-0.0648
	(0.1420)	(0.1576)	(0.2972)
**LnperGDP**	0.4048***	0.3909***	0.9987***
	(0.0245)	(0.0270)	(0.0469)
**Constant**	-14.7527***	-14.8067***	-16.5849***
	(0.3456)	(0.3812)	(0.6370)
**Observations**	22,492	18,437	18,941
**R-squared**	0.3620	0.3656	-

**Notes:** model (1) is the parameter estimation of the enterprise’s financialization behavior; model (2) is the parameter estimation after the original enterprise’s financialization behavior is replaced by the financialization income, and model (3) is the parameter estimation using GMM method; in brackets are the robust standard errors of the corresponding parameters;

* * * represents P < 0.01;

* * represents P < 0.05;

* represents P < 0.1.

The results of parameter estimation are reported in Table 3. The coefficient of enterprise financialization in Column (1) is -0.3694 (P < 0.01). At the same time, Columns (2) and (3) in Table 3 respectively present the parameter estimation results of financialization substitution variables and the GMM method. The sign of the parameter estimation results is consistent with that in Column (1), with slight differences in the absolute value and significance of the coefficients, indicating that the estimation results are robust. It can be seen from the empirical results that the regression coefficients of corporate financialization behavior pass the significance level of 5% and show negative effects whether changing the explanatory variables or the estimation method of the model. These results indicate that enterprise financialization has a significant negative impact on technological innovation, that is, the higher the degree of enterprise financialization is, the more strongly the level of technological innovation will be inhibited. From the perspective of control variables, the coefficients of enterprise age, capital intensity, and deposit loan ratio are significantly negative at the level of 1%, indicating that these factors have an inhibitory effect on technological innovation; the coefficients of operating net cash flow, enterprise scale, industrial structure, and economic development level of the region where an enterprise is located are significantly positive at the 1% level, indicating that these factors are conducive to promoting the technological innovation of enterprises. It can be known from the above analysis that the financialization behavior of enterprises has an inhibiting effect on technological innovation, which verifies Hypothesis 1.

### Econometric test of the impact mechanism of enterprise financialization on technological innovation

According to the theoretical analysis and Hypothesis 2 in the second part, this paper adopts the sequential test method to examine the impact mechanism of enterprise financialization on technological innovation step by step.

First of all, based on the benchmark Model (1), the comprehensive effect of financialization behavior on technological innovation is tested without adding mediating variables to the benchmark model.

Secondly, taking the mediating variables as the explained variables and the enterprise financialization behavior as the core explanatory variable, this paper identifies whether the financialization behavior has an impact on the mediating variables. The specific model forms are as follows:

$${\text{Lev}}_{\text{it}}=\beta_{0}+\beta_{1}{\text{Financialzation}}_{\text{it}}+\sum \alpha_{\text{i}}{\text{X}}_{\text{it}}+\varepsilon_{\text{it}},$$ (2)

$${\text{Roa}}_{\text{it}}=\beta_{0}+\beta_{1}{\text{Financialzation}}_{\text{it}}+\sum \alpha_{\text{i}}{\text{X}}_{\text{it}}+\varepsilon_{\text{it}},$$ (3)

Finally, based on the benchmark model, the mediating variables (Lev and Roa) are included in the benchmark model to test whether the financialization behavior of enterprises affects the technological innovation of enterprises by influencing the capital structure and the net profit margin of enterprises’ operations. The specific model forms are shown in Eqs (4) and (5).


$$\text{LnApply}\_\text{sit}=\beta_{0}+{\eta \text{Financialzation}}_{\text{it}}+{\theta \text{Lev}}_{\text{it}}+\sum \alpha_{\text{i}}{\text{X}}_{\text{it}}+\varepsilon_{\text{it}},$$ (4)

$$\text{LnApply}\_\text{sit}=\beta_{0}+{\eta \text{Financialzation}}_{\text{it}}+{\theta \text{Roa}}_{\text{it}}+\sum \alpha_{\text{i}}{\text{X}}_{\text{it}}+\varepsilon_{\text{it}},$$ (5)

The subscripts i and t in models (2), (3), (4), and (5) denote enterprise and year, respectively, and the meanings of other variables are completely consistent with those in Table 1.

### Empirical analysis of the impact mechanism of enterprise financialization on technological innovation

By estimating the parameters of Models (2), (3), (4) and (5) and analyzing the significance of the estimated coefficients β_1_, η, θ, we can judge whether the relevant variables have a mediating effect and the level. Based on this, the parameter estimation results of the capital structure and the net profit margin of the operation of the enterprise are respectively shown in Table 4.

**Table 4:** Mediating effect tests of Lev and Roa.

Variable	Lev	LnApply_s	Roa	LnApply_s
	(1)	(2)	(3)	(4)
**Financialization**	-0.2631***	-0.5383***	-0.0020	-0.3676***
	(0.0102)	(0.0925)	(0.0043)	(0.0903)
**Lev**		-0.5373***		
		(0.0606)		
**Roa**				0.8807***
				(0.1889)
**CFO**	-0.4446***	0.0128	0.3451***	-0.0588
	(0.0169)	(0.1429)	(0.0079)	(0.1531)
**Lnsize**	0.0798***	0.5449***	0.0007*	0.5015***
	(0.0010)	(0.0104)	(0.0004)	(0.0091)
**Lnage**	0.0816***	-0.0854***	-0.0170***	-0.1224***
	(0.0038)	(0.0329)	(0.0013)	(0.0328)
**Fixed**	0.1139***	-1.4718***	-0.0947***	-1.4325***
	(0.0083)	(0.0714)	(0.0029)	(0.0722)
**Loandep**	0.0301***	-0.3660***	-0.0047	-0.3745***
	(0.0111)	(0.0968)	(0.0046)	(0.0961)
**Industr**	0.0451***	1.5851***	0.0221***	1.5356***
	(0.0166)	(0.1439)	(0.0070)	(0.1420)
**LnperGDP**	-0.0255***	0.3941***	0.0090***	0.3970***
	(0.0029)	(0.0248)	(0.0010)	(0.0245)
**Constant**	-1.2569***	-15.4679***	-0.0087	-14.7450***
	(0.0409)	(0.3546)	(0.0165)	(0.3451)
**Observations**	22,084	22,084	22,492	22,492
**R-squared**	0.4073	0.3660	0.1822	0.3628

**Note:** Columns (1), (2), (3) and (4) are the estimated results of Eqs (2), (4), (3) and (5), respectively; the robust standard errors of the corresponding parameters are in parentheses.

*** means p<0.01,

** means p<0.05, and

* means p<0.1.

It can be seen from Table 4 that the mediating effect of Lev (the capital structure of enterprises) is significant. The effect of enterprise financialization behavior on the mediating variable Lev is -0.2631 (P <0.01). After controlling the influence of other factors, the effect of the mediating variable Lev on technological innovation is -0.5373 (P <0.01). Therefore, the mediating effect of enterprise financialization behavior on technological innovation through Lev is (-0.2631)×(-0.5373) = 0.1414. The total effect of enterprise financialization behavior on technological innovation is -0.3694 (P <0.01), so the mediating rate is 26.26%. Since the effect of enterprise financialization behavior on the mediating variable Roa (the net profit margin of enterprises) is not significant, the Bootstrap method should be adopted for further testing, and the test results are shown in Table 5.

**Table 5:** Test results of the mediating effect of Roa on technological innovation with the Bootstrap method.

	Observed Coef.	Bias	Std. Err.	[95% Conf. Interval]		
**Indirect effect**	-0.0093	-0.0007	0.0081	-0.0274	0.0053	(P)
				-0.0264	0.0058	(BC)
**Direct effect**	-1.2535	0.0004	0.0996	-1.4533	-1.0533	(P)
				-1.4534	-1.0546	(BC)

**Note:** (P) represents the percentage position confidence interval; (BC) represents the deviation correction confidence interval.

It can be seen from Table 5 that the indirect effect of Roa on technological innovation is not significant, that is, there is no mediating effect. Combined with Tables 4 and 5, the hypothesis about the impact mechanism of financialization on technological innovation (Hypothesis 2) is verified.

## Heterogeneity of the impact of enterprise financialization on technological innovation

Although the above results on the whole verify the impact of enterprise financialization on technological innovation, their relationship is also affected by many factors which deserve consideration. Therefore, this section considers the internal attributes and external impacts and analyzes the differences of the impact of enterprise financialization on technological innovation according to different characteristics of enterprise attributes and external environment, so as to reveal the heterogeneity of the financialization impact of different types of enterprises on their technological innovation.

### Heterogeneity analysis based on attribute characteristics

Due to the different attributes of enterprises, the impact of their financialization behaviors on technological innovation may be different. From the perspective of industry characteristics of enterprises, enterprises in industries with different pollution levels have different levels of pollution to the environment and different pollution treatment costs. Therefore, their financialization behaviors have different impacts on technological innovation. Enterprises in heavily polluting industries have a large degree of environmental pollution, and the treatment of environmental pollution mainly depends on technological innovation [34], but the treatment cost is relatively high. Enterprises in non-heavy pollution industries have a lower degree of environmental pollution and lower cost of treatment. Therefore, the two types of enterprises have different views and motivations on technological innovation. Based on this, according to the pollution characteristics of the industry to which the enterprise belongs, the enterprise sample is divided into heavy pollution and non-heavy pollution industries two sub-samples (Classification of heavy pollution and non-heavy pollution industries: according to the “Industry Classification Guidelines of Listed Companies” revised by China Securities Regulatory Commission in 2012, and“The Catalogue of Classified Management of Environmental Protection Inspection Industry of Listed Companies” and “The Guide to Environmental Information Disclosure of Listed Companies” formulated by the Ministry of Environmental Protection in 2008, the heavy pollution industries in this paper include: coal mining and washing industry, oil and gas mining industry, ferrous metal mining and dressing industry, non-ferrous metal mining and dressing industry, textile industry Leather, fur, feather and their products and shoemaking industry, paper and paper products industry, petroleum processing, coking and nuclear fuel processing industry, chemical raw materials and chemical products manufacturing industry, pharmaceutical manufacturing industry, chemical fiber manufacturing industry, non-metallic mineral products industry, ferrous metal smelting and rolling processing industry, non-ferrous metal smelting and rolling processing industry, metal products industry, electric power, thermal power production and processing industry Supply industry (16 categories); other industries are non-heavy pollution industries.).

Due to different ownership characteristics of enterprises, the impact of their financialization on technological innovation may be different. There are many types of ownership enterprises in China, but state-owned enterprises, private enterprises, and foreign-funded enterprises are the main. There are some differences in resource endowment among different types of ownership, especially in the aspects of enterprise scale, capital, talent, technical level, and economic policy. Therefore, the impact of their financialization on technological innovation may be different. Based on this, according to the different ownership structure, this paper divides the enterprise sample into three sub-samples: state-owned enterprises, private enterprises, and other enterprises.

Based on the above theoretical analysis, the whole sample is divided into sub-samples according to industry attributes and ownership attributes. The panel regression model is further adopted to study the heterogeneity of the impact of enterprise financialization on technological innovation, and the results of parameter estimation are shown in Table 6.

**Table 6:** Test results of the impact of enterprise financialization on technological innovation based on enterprise attributes.

	According to industry attributes		According to ownership attributes		
variable	Heavy pollution industry	Non-heavy pollution industry	state-owned	private	others
	(1)	(2)	(3)	(4)	(5)
**Financialization**	-0.2586	-0.3466***	-0.2274	-0.0294	-1.8949***
	(0.1913)	(0.1005)	(0.1433)	(0.1268)	(0.3274)
**CFO**	0.8823***	0.1554	0.1835	0.2611	0.7857
	(0.2539)	(0.1634)	(0.2181)	(0.1944)	(0.5430)
**Lnsize**	0.5230***	0.5326***	0.4948***	0.5263***	0.5052***
	(0.0151)	(0.0111)	(0.0134)	(0.0146)	(0.0373)
**Lnage**	-0.2518***	-0.0497	-0.0934*	-0.1605***	-0.3628***
	(0.0599)	(0.0378)	(0.0518)	(0.0451)	(0.1368)
**Fixed**	-1.2334***	-0.9482***	-2.0711***	-0.9045***	-0.7153***
	(0.1203)	(0.0879)	(0.0972)	(0.1140)	(0.2720)
**Loandep**	-0.0869	-0.4861***	0.0081	-0.5936***	-0.8582**
	(0.1627)	(0.1156)	(0.1539)	(0.1307)	(0.3998)
**Industr**	0.0220	2.0406***	1.1430***	1.5667***	3.1503***
	(0.2677)	(0.1674)	(0.2063)	(0.2076)	(0.6037)
**LnperGDP**	0.2688***	0.3731***	0.4167***	0.3774***	0.3832***
	(0.0411)	(0.0302)	(0.0361)	(0.0358)	(0.1218)
**Constant**	-11.6648***	-15.6293***	-14.8309***	-14.1711***	-14.0861***
	(0.5591)	(0.4208)	(0.5047)	(0.5246)	(1.6352)
**Observations**	6,485	16,007	9,114	11,810	1,568
**R-squared**	0.2768	0.4268	0.4506	0.3015	0.3304

**Note:** The robust standard errors of the corresponding parameters are in brackets;

*** means p<0.01,

** means p<0.05, and

* means p<0.1.

Table 6 reports the differences in the impact of financialization on technological innovation among different types of enterprises. In the group regression of non-heavily polluting industries and heavily polluting industries, the impact of enterprise financialization on technological innovation is heterogeneous, and the regression coefficients of enterprise financialization behavior are -0.2586 and -0.3466, respectively. The financialization behavior of enterprises in non-heavily polluting industries has a significant inhibitory effect on technological innovation, which may be due to that although enterprises of non-heavily polluting industries have relatively small degree of environmental pollution, their pollution is mainly solved through technological innovation, and pollution control is a long-term process, facing greater uncertainty, therefore, managers tend to prefer financial investment with short cycle and fast income, which inhibits the level of technological innovation of enterprises in non-heavily polluting industries. However, there is not enough evidence to prove that the financialization, behavior of enterprises in heavily polluting industries has an impact on their technological innovation.

In the group regression of state-owned enterprises, private enterprises and other enterprises, the influence of enterprise financialization on technological innovation is heterogeneous, and the regression coefficients of enterprise financialization behavior are -0.2274, -0.0294 and -1.8949, respectively, indicating that the financialization behavior of other enterprises has a significant inhibiting effect on the technological innovation. According to Schumpeter’s theory, innovation requires a relatively relaxed environment, and large enterprises and those with market monopolies are more capable and motivated to innovate. Other enterprises have relatively strong internal characteristics and target heterogeneity, and they are weak in risk tolerance of technological innovation. Therefore, they are more inclined to short-term gains brought by financialized investment, which is often not conducive to the improvement of technological innovation ability. However, there is no sufficient evidence to prove that the financialization behavior of state-owned enterprises and private enterprises has an impact on their technological innovation.

Further analysis in combination with Table 3 shows that, in the case of the full sample, the financialization regression coefficient is -0.3694. Based on this coefficient, the degree to which different types of sub-samples deviate from the full sample can be calculated. From the perspective of the industry attribute of the enterprise, the downward deviation of the non-heavily polluting industry is 6.17% ((0.3466–0.3694)/0.3694 = -6.17%, and the subsequent calculation method is the same). From the perspective of the ownership structure of enterprises, other enterprises deviated upward by 412.97%. It can be seen that the ownership attribute has a greater impact on the relationship between enterprise financialization and technological innovation.

### Heterogeneity analysis based on the external environment

The impact of enterprise financialization behaviors on technological innovation may be different due to different regions where enterprises are located, for in different regions, there are differences in geographical location, history, social economy, cultural environment, political and legal factors. The eastern region in China is the region with the fastest economic development and has advantages over the central and western regions in terms of economic development level, science and technology, which can provide a good environment for technological innovation of enterprises. The economic development in western China started late, the speed is relatively slow, and the conditions for enterprise technological innovation are limited, which affects enterprise technological innovation activities to a certain extent. Based on this, this paper divides the enterprises into three sub-samples: eastern enterprises, central enterprises, and western enterprises according to the different regions where the enterprises are located.

Due to different financing constraints of enterprises, the impact of their financialization behaviors on technological innovation may be different. Innovation is a cumulative, collaborative and uncertain process that requires a large amount of financial support. Therefore, innovation activities of enterprises are affected by financing constraints to some extent [35, 36]. At the same time, the financial behavior of enterprises is also affected by the degree of enterprise capital adequacy, which affects the technological innovation activities of enterprises. Based on this, according to the financing constraints faced by enterprises, the sample enterprises are divided into two sub-samples: high financing constraint enterprises and low financing constraint enterprises (Division of financing constraints: enterprise scale is used as the proxy variable to measure the intensity of financing constraints. Low enterprise scale indicates high financing constraints; on the contrary, it indicates low financing constraints. According to the median size of enterprises, the top 50% of enterprises are high financing constraint enterprises, while the bottom 50% are low financing constraint enterprises.) to perform a heterogeneous analysis.

Based on the above theoretical analysis, the whole sample is divided into sub-samples according to the region where the enterprise is located and the degree of financing constraint the enterprise faces. According to the region where the enterprise is located, it can be divided into eastern enterprises, central enterprises and western enterprises. According to the degree of financing constraint, it can be divided into high financing constraint enterprises and low financing constraint enterprises. The panel regression model is further adopted to study the heterogeneity of the impact of enterprise financialization on technological innovation, and the results of parameter estimation are shown in Table 7.

**Table 7:** Test results of the impact of enterprise financialization on technological innovation based on the external environment.

	According to the region where the enterprise is located			According to the degree of financing constraint	
**Variable**	eastern	central	western	high financing constraint	low financing constraint
	(1)	(2)	(3)	(4)	(5)
**Financialization**	-0.3339***	-0.2830	-0.5628**	-0.3076***	-0.5242***
	(0.1046)	(0.2484)	(0.2536)	(0.1155)	(0.1439)
**CFO**	0.4218**	-0.2655	-0.2899	-0.1085	0.6950***
	(0.1699)	(0.3395)	(0.3505)	(0.1760)	(0.2174)
**Lnsize**	0.5160***	0.4828***	0.5073***	0.6129***	0.5168***
	(0.0112)	(0.0216)	(0.0235)	(0.0228)	(0.0176)
**Lnage**	-0.0105	-0.4242***	-0.2194**	-0.2672***	-0.0427
	(0.0391)	(0.0816)	(0.0916)	(0.0421)	(0.0489)
**Fixed**	-1.3271***	-1.5833***	-1.9453***	-1.1244***	-2.0498***
	(0.0906)	(0.1490)	(0.1616)	(0.0938)	(0.1027)
**Loandep**	-0.5778***	0.4260	-0.3629*	-0.3900***	-0.4452***
	(0.1226)	(0.4109)	(0.2107)	(0.1253)	(0.1436)
**Industr**	2.0044***	1.8981***	3.6843***	2.2195***	0.9373***
	(0.1855)	(0.4620)	(0.7001)	(0.1833)	(0.2125)
**LnperGDP**	0.5500***	-0.7681***	-0.6228***	0.3549***	0.4781***
	(0.0518)	(0.1677)	(0.1291)	(0.0314)	(0.0376)
**Constant**	-16.9599***	-3.4134*	-5.7520***	-16.0977***	-15.8931***
	(0.6238)	(1.7696)	(1.1330)	(0.5902)	(0.6042)
**Observations**	15,227	3,952	3,313	11,774	10,718
**R-squared**	0.3627	0.3878	0.3823	0.2658	0.4288

**Note**: The robust standard errors of the corresponding parameters are in brackets;

*** means p<0.01,

** means p<0.05, and

* means p<0.1.

Table 7 reports the impact of financialization in different types of enterprises on technological innovation. In the group regression of different regions, the impact of enterprise financialization on technological innovation is heterogeneous, and the regression coefficients of enterprise financialization are -0.3339, -0.2830, and -0.5628, respectively. The financialization behavior of eastern enterprises and western enterprises has an inhibitory effect on technological innovation, and the inhibitory effect of the latter is greater than that of the former. The reason may be that the eastern region provides better conditions for enterprises’ technological innovation activities than the western region, and the development of innovation activities is relatively smooth, while the western enterprises are more inclined to choose financial investment due to the constraints of the external environment, so the inhibition of technological innovation activities is stronger. However, there is not enough evidence to prove that the financialization behavior of central enterprises has a significant impact on technological innovation.

In the group regression of different financing constraints, the impact of enterprise financialization on technological innovation is heterogeneous, and the regression coefficients of enterprise financialization are -0.3076 and -0.5242, respectively. The financial behaviors of enterprises with both high and low financing constraints have a significant inhibitory effect on their technological innovation, and the inhibitory effect on the latter is greater than that of the former. The reasons are as follows: the financing ability of enterprises with high financing constraints is weak, and the funds used for financial investment are relatively limited; while the financing ability of enterprises with low financing constraints is strong, the funds of enterprises are relatively abundant, and the funds used for financial investment are more, so the inhibition intensity of technological innovation is also greater.

Similarly, further analysis in combination with Table 3 shows that, in the case of the full sample, the financialization regression coefficient is -0.3694. Based on this coefficient, the degree to which different types of sub-samples deviate from the full sample can be calculated. From the point of view of the region where the enterprises are located, the eastern enterprises deviate downward by 9.61%, and the inhibition of financialization on technological innovation is weaker than the average level. The upward deviation of enterprises in western China is 52.36%, and the inhibition of financialization on technological innovation is much stronger than the average level. From the perspective of financing constraints faced by enterprises, high financing constraints enterprises deviate downward by 16.73%, while low financing constraints enterprises deviate upward by 41.91%. Therefore, it can be seen that the region and financing constraints have similar effects on the relationship between enterprise financialization and technological innovation. Based on the above analysis, the heterogeneity hypothesis (Hypothesis 3) about the impact of financialization behavior on technological innovation has been verified.

## Results

This paper selects non-financial enterprises of China’s A-share listed companies from 2007 to 2017 as the research object. Panel regression model and mediating model are employed to study the impact of enterprise financialization on technological innovation, its impact mechanism, and the heterogeneity under different enterprise attributes and external environment. The main conclusions are as follows.

First, the financialization behavior of enterprises has a significant "crowding-out" effect on technological innovation. This paper conducts an empirical study on the sample of non-financial listed companies in China and finds that financialization has a significant negative effect on technological innovation. This paper verifies that after the 2008 financial crisis, the dual effects of the goal of maximizing shareholder value and the decline of marginal profit of the real economy are strengthened, making the enterprise financialization exert a significant crowding out effect on technological innovation.

Second, enterprise financialization mediates through capital structure rather than performance. Although the goal of enterprise financialization behavior is to maximize enterprise value and performance, in the impact mechanism of financialization on technological innovation, the mediating effect is not through performance variables but through capital structure such as the leverage ratio. In this paper, the leverage ratio represents the capital structure of the enterprise, and the net profit of the enterprise is screened as the performance mediating variable. Through the empirical analysis of the sample data, it is found that the capital structure variable has the mediating effect, but the performance index does not. Based on this, the financialization behavior of enterprises is more inclined to the use of financial assets, and it is not necessarily able to achieve the goal of maximizing shareholder value in terms of operational orientation.

Third, enterprises have different attributes and face different external environment, so the impact of financialization on technological innovation is heterogeneous. The impact of enterprise financialization on technological innovation is closely related to the attribute characteristics of enterprises, and there is heterogeneity among enterprises with different attribute characteristics. For enterprises in industries with different pollution levels, the financialization of enterprises in non-heavy pollution industries has a significant inhibitory effect on technological innovation, but the financialization of enterprises in heavy pollution industries has no significant effect on technological innovation. For enterprises with different ownership attributes, the financialization of other enterprises has a significant inhibitory effect on technological innovation, but the financialization of state-owned enterprises and private enterprises has no significant effect on technological innovation. The impact of enterprise financialization on technological innovation is also closely related to the external environment faced by enterprises, and there is heterogeneity among enterprises in different external environments. For different enterprises in different regions, the effect of financialization of western enterprises on technological innovation is stronger than that of eastern enterprises, but the effect of financialization of central enterprises on technological innovation is not significant. For enterprises with different financing constraints, the effect of financialization of enterprises with low financing constraints on technological innovation is significantly greater than that of enterprises with high financing constraints.

Fourth, there are significant differences in the impact of financialization on technological innovation between enterprise attributes and their external environment. Through the sub-sample study, the two types of attribute characteristics and two kinds of external environment selected in this paper affect the relationship between enterprise financialization and technological innovation. Among them, the deviation of external environment from the relationship between enterprise financialization and technological innovation is not more than 55%, while the deviation of enterprise attribute characteristics from the whole sample is more than 400%. It can be seen that the relationship between enterprise financialization and technological innovation is more easily affected by the characteristics of enterprise attributes.

## Implications

Combined with the above research conclusions, this paper puts forward the following implications. First, the high yield in the financial industry is the motivation for listed companies to enter this field to absorb excess profits, so the government should relax the industry access, encourage healthy competition among industries, break the monopoly of the financial industry, and promote the profit balance among industries, so as to enhance the power of enterprises in technological innovation. Second, the government should start from both the internal and external aspects of enterprises and formulate different supporting policies in combination with the attributes and external environment of enterprises, so as to provide enterprises with conditions for technological innovation and actively guide them to carry out technological innovation activities. Third, the government should increase the support for the innovation and development of those non-heavy pollution enterprises with development potential and high technology content, so as to alleviate the uncertainty in the period of transformation and upgrading. Enterprises with financing problems should also be paid attention to and helped to increase R & D investment by reducing financing costs, expanding financing channels, and adding and deducting R & D investment. In addition, means like reducing the tax burden and technical subsidies can also be used to improve the profit margin of enterprises.

This paper empirically studies the mechanism and heterogeneity of non-financial enterprises’ financialization behavior affecting technological innovation. However, due to the limited samples and periods, there are certain limitations, which are reflected in two aspects. On the one hand, the corporate social responsibility issues are not included. In the process of financialization, enterprises should not only take responsibility to employees’ welfare, but more importantly, they must bear environmental responsibility. Accordingly, enterprises should pay more attention to green technology innovation in the process of R & D innovation. However, this research does not take this aspect into consideration. On the other hand, this paper doesn’t pay enough attention to the identification of enterprise financialization behavior. Enterprise financialization has a heterogeneous impact on technological innovation, but this impact may have dynamic characteristics. Besides, different non-financial enterprises have different attributes, which make the allocation of financial assets dynamic, and there might be an appropriate range. The study doesn’t identify the financialization behavior according to this appropriate range.

According to the above mentioned limitations, there is room for further research on the following issues. The first is the issues related to corporate environmental responsibility. For corporate environmental responsibility, the research contents include the measurement of corporate environmental responsibility and the influence of corporate financialization behavior on corporate environmental responsibility. The second is the research on the appropriate range of the financialization level of enterprises. According to the selected samples, the corresponding target optimization criteria of non-financial enterprises can be set, and the appropriate range of the financialization level of non-financial enterprises can be obtained through the intersection of the optimal criteria of different targets.
